# Antimicrobial activities of flavonoid glycosides from *Graptophyllum grandulosum* and their mechanism of antibacterial action

**DOI:** 10.1186/s12906-018-2321-7

**Published:** 2018-09-15

**Authors:** Cyrille Ngoufack Tagousop, Jean-de-Dieu Tamokou, Steve Endeguele Ekom, David Ngnokam, Laurence Voutquenne-Nazabadioko

**Affiliations:** 1 Research Unit of Environmental and Applied Chemistry, Department of Chemistry, Faculty of Science, University of Dschang, P.O. Box 67, Dschang, Cameroon; 2 Research Unit of Microbiology and Antimicrobial Substances, Department of Biochemistry, Faculty of Science, University of Dschang, P.O. Box 67, Dschang, Cameroon; 3 Groupe Isolement et Structure, Institut de Chimie Moléculaire de Reims (ICMR), CNRS UMR 7312, Bat. 18 BP.1039, 51687 Reims cedex 2, France

**Keywords:** *Graptophyllum glandulosum*, Acanthaceae, Flavonoid glycosides, Antibacterial, Antifungal, Mode of action

## Abstract

**Background:**

The search for new antimicrobials should take into account drug resistance phenomenon. Medicinal plants are known as sources of potent antimicrobial compounds including flavonoids. The objective of this investigation was to evaluate the antimicrobial activities of flavonoid glycosides from *Graptophyllum grandulosum*, as well as to determine their mechanism of antibacterial action using lysis, leakage and osmotic stress assays.

**Methods:**

The plant extracts were prepared by maceration in organic solvents. Column chromatography of the *n*-butanol extract followed by purification of different fractions led to the isolation of five flavonoid glycosides. The antimicrobial activities of extracts/compounds were evaluated using the broth microdilution method. The bacteriolytic activity was evaluated using the time-kill kinetic method. The effect of extracts on the red blood cells and bacterial cell membrane was determined by spectrophotometric methods.

**Results:**

Chrysoeriol-7-*O*-*β*-D-xyloside (**1**), luteolin-7-*O*-*β*-D-apiofuranosyl-(1 → 2)-*β*-D-xylopyranoside **(2**), chrysoeriol-7-*O*-*β*-D-apiofuranosyl-(1 → 2)-*β*-D-xylopyranoside (**3**), chrysoeriol-7-*O*-*α*-L-rhamnopyranosyl-(1 → 6)-*β*-D-(4"-hydrogeno sulfate) glucopyranoside (**4**) and isorhamnetin-3-*O*-*α*-L-rhamnopyranosyl-(1 → 6)-*β*-D-glucopyranoside (**5**) were isolated from *G. grandulosum* and showed different degrees of antimicrobial activities. Their antibacterial activities against multi-drug-resistant *Vibrio cholerae* strains were in some cases equal to, or higher than those of ciprofloxacin used as reference antibiotic. The antibacterial activities of flavonoid glycosides and chloramphenicol increased under osmotic stress (5% NaCl) whereas that of vancomycin decreased under this condition. *V. cholerae* suspension treated with flavonoid glycosides, showed a significant increase in the optical density at 260 nm, suggesting that nucleic acids were lost through a damaged cytoplasmic membrane. A decrease in the optical density of *V. cholerae* NB2 suspension treated with the isolated compounds was observed, indicating the lysis of bacterial cells. The tested samples were non-toxic to normal cells highlighting their good selectivity index.

**Conclusions:**

The results of the present study indicate that the purified flavonoids from *G. glandulosum* possess antimicrobial activities. Their mode of antibacterial activity is due to cell lysis and disruption of the cytoplasmic membrane upon membrane permeability.

## Background

The development of resistance by microorganisms to existing antimicrobial agents has been known for a long time. In several findings, the emergence of multidrug resistant strains of *Vibrio cholerae* O1 and *Shigella flexneri* has been reported due to different genetic factors including transfer of plasmids, integrons and allelic variation in the specific genes [1]. In developing countries, fluoroquinolones, widely used for the treatment of many bacterial diseases, including cholera and shigellosis, could contribute to the emergence of multidrug resistance among potential enteric pathogens. Although significant progress has been made in microbiological research and in the control of many diseases caused by infectious microorganisms, recurrent epidemics due to drug-resistant microorganisms as well as the appearance of new microbial pathogenic strains demand the discovery of new antibiotics. The need for ecologically safe compounds as therapeutic agents against drug-resistant microorganisms has driven many studies toward medicinal plants. Literature shows thousands of plant species that have been tested in vitro against many fungal and bacterial strains, and a good number of medicinal plant extracts and pure compounds have now been proven to be active against fungi, Gram-positive and Gram-negative bacteria [2–5]. Medicinal plants contain therapeutic amount of secondary metabolites including flavonoids. These are polyphenolic and C_6_-C_3_-C_6_ compounds in which the two C_6_ groups are substituted benzene rings, and the C_3_ is an aliphatic chain which contains a pyran ring [6]. They occur as *O*- or *C*-glycosides or in the free state as aglycones with hydroxyl or methoxyl groups [7]. The sugar moiety is an important factor determining their bioavailability. Flavonoids may be divided into seven types: flavones, flavonols, flavonones, flavanes, isoflavones, biflavones and chalcones [8]. Flavonoids are well documented for their pharmacological effects, including antimicrobial, anticancer, antiviral, antimutagenic and antiinflammatory activities [9–11]. Biological properties of flavonoids are linked to their ability to act as strong antioxidants and free radical scavengers, to chelate metals, and to interact with enzymes, adenosine receptors and biomembranes [7].

*Graptophyllum glandulosum* Turrill (Acanthaceae) is a shrub with 4-angled, nearly glabrous branches, normal green leaves, and red-purple flowers 1 in. or more long. It is one of several shrubs and trees of *Graptophyllum* that mainly grow in West and Central Africa but also in the pacific regions [12]. This plant contains some important secondary metabolites such as polyphenols, flavonoids and glycosides [13]. Leaves, roots and other parts of *G. glandulosum*, are used in folk medicine in Cameroon to treat wounds, abscesses, skin diseases, respiratory tract infections and diarrhea. Medical importance of this plant attracted us to explore its antimicrobial properties. Although several ethnobotanical reports have emphasized the pharmacological importance of this species for conditions that appear to be associated with microbial infections, there is very limited literature concerned with the identification of the antimicrobial compounds from this plant. However, a few reports on the in vitro antimicrobial activity of plants have been published [14, 15]. The objective of this investigation was to evaluate the antimicrobial activities of flavonoid glycosides from *G. grandulosum*, as well as to determine their mechanism of action using lysis, leakage, and osmotic stress assays.

## Methods

### General experimental procedures

#### Melting point

A Schorpp Gerätetechnik (Germany) apparatus was used to take the melting points of different compounds.

#### NMR analysis

The 1D (^1^H and ^13^C-NMR) and 2D (COSY, NOESY, HSQC and HMBC) spectra were performed in deuterated solvents (CD_3_OD) on Bruker Avance III 600 spectrometer at 600 MHz/150 MHz. All chemical shifts (*δ*) are given in ppm units with reference to tetramethylsilane (TMS) as internal standard and the coupling constants (*J*) are in Hz.

#### Spectrometric analysis

The mass spectra **(**HR-TOFESIMS) were carried out on Micromass Q-TOF micro instrument (Manchester, UK). Samples were introduced by direct infusion in a solution of MeOH at a rate of 5 μL/min.

#### Chromatographic methods

Silica gel 60 Merck, 70–230 mesh and sephadex LH-20 were used to perform column chromatography while precoated silica gel 60 F_254_ (Merck) plates, were used to perform thin layer chromatography. The spots were visualized by an UV lamp multiband UV-254/365 nm (ModelUVGL-58 Upland CA 91786, U.S.A) followed by spraying with 50% H_2_SO_4_ and heating at 100 °C for 5 min.

#### Plant material

The aerial parts of *G. grandulosum* were harvested in a small village called Foto situated in the Menoua Division, Western region of Cameroon) in November 2015. The Plant was identified and authenticated by a Cameroonian Botanist (Mr. Fulbert Tadjouteu) at the National Herbarium where a voucher specimen was archived (N^o^ 65631/HNC).

#### Extraction and isolation

The extraction and isolation of compounds were done as previously described [13]. Briefly, the aerial part of *G. grandulosum* was air-dried and powdered. The powder was macerated at room temperature with MeOH to afford the MeOH extract. Part of this extract (235 g) was suspended in water (300 mL) and successively partitioned with EtOAc and *n*-BuOH to yield 37 and 13 g of extracts, respectively. Column chromatography of the *n*-BuOH extract followed by purification of different fractions led to the isolation of five compounds.

#### Structural identification of the isolated compounds

The structures of isolated compounds were determined after interpretation of their physical, spectrometric and spectroscopic data summarized in this subsection.

Chrysoeriol-7-*O*-*β*-D-xyloside (**1):** yellow amorphous powder; molecular formula C_21_H_20_O_10_; ^13^C NMR (CD_3_OD, 150 MHz) *δ*_*C*_: 165.3 (C-2), 104.3 (C-3), 184.1 (C-4), 161.7 (C-5), 99.6 (C-6), 163.1 (C-7), 95.8 (C-8), 158.5 (C-9), 107.0 (C-10), 123.1 (C-1′), 110.5 (C-2′), 148.2 (C-3′), 151.0 (C-4′), 116.7 (C-5′), 121.9 (C-6′), 55.2 (C-7′) for aglycone; 100.9 (C-1′′), 74.4 (C-2′′), 77.3 (C-3′′), 70.7 (C-4′′), 66.9 (C-5′′) for sugar moiety. ^1^H NMR data (CD_3_OD, 600 MHz) *δ*_*H*_: 6.62 (1H, s, H-3), 6.38 (1H, d, *J* = 2.1 Hz, H-6), 6.71 (1H, d, *J* = 2.1 Hz, H-8), 7.44 (1H, d, *J* = 2.1 Hz, H-2′), 6.84 (1H, d, *J* = 8.4 Hz, H-5′), 7.47 (1H, dd, *J* = 8.4 and 2.1 Hz, H-6′), 3.87 (3H, s, H-7′) for aglycone; 4.95 (1H, d, *J* = 7.1 Hz, H-1′′), 3.37 (1H, m, H-2′′), 3.36 (1H, m, H-3′′), 3.49 (1H, m, H-4′′), 3.38 (1H, m, H-5′′a), 3.87 (1H, m, H-5′′b) for sugar moiety.

Luteolin-7-*O*-*β*-D-apiofuranosyl-(1 → 2)-*β*-D-xylopyranoside (**2):** yellow powder; molecular formula C_25_H_26_O_14_. m.p. = 203 °C. ^13^C NMR data (CD_3_OD, 150 MHz) *δ*_*C*_: 165.3 (C-2), 103.5 (C-3), 182.5 (C-4), 162.9 (C-5), 99.7 (C-6), 163.1 (C-7), 94.9 (C-8), 157.4 (C-9), 105.5 (C-10), 121.7 (C-1′), 114.2 (C-2′), 146.4 (C-3′), 150.5 (C-4′), 116.6 (C-5′), 119.7 (C-6′) for aglycone; 99.0 (C-1′′), 76.0 (C-2′′), 77.0 (C-3′′), 70.0 (C-4′′), 66.1 (C-5′′), 109.3 (C-1′′′), 76.5 (C-2′′′), 79.7 (C-3′′′), 64.5 (C-4′′′), 74.4 (C-5′′′) for sugar moiety. ^1^H NMR data (CD_3_OD, 600 MHz) *δ*_*H*_: 6.75 (1H, s, H-3), 6.40 (1H, d, *J* = 2.1 Hz, H-6), 6.75 (1H, d, *J* = 2.1 Hz, H-8), 7.44 (1H, d, *J* = 2.1 Hz, H-2′), 6.90 (1H, d, *J* = 8.4 Hz, H-5′), 7.47 (1H, dd, *J* = 8.4 and 2.1 Hz, H-6′) for aglycone; 5.18 (1H, d, *J* = 7.1 Hz, H-1′′), 3.52 (1H, dd, *J* = 9.0 and 7.1 Hz, H-2′′), 3.43 (1H, m, H-3′′), 3.41 (1H, m, H-4′′), 3.78 (1H, dd, *J* = 9.7 and 3.4 Hz, H-5′′a), 3.42 (1H, dd, *J* = 9.7 and 3.4 Hz, H-5′′b), 5.34 (1H, d, *J* = 1.3 Hz, H-1′′′), 3.75 (1H, m, H-2′′′), 3.30 (2H, d, *J* = 3.4 Hz, H-4′′′), 3.88 (1H, d, *J* = 9.3 Hz, H-5′′′a), 3.65 (1H, d, *J* = 9.3 Hz, H-5′′′b) for sugar moiety.

Chrysoeriol-7-*O*-*β*-D-apiofuranosyl-(1 → 2)-*β*-D-xylopyranoside (**3):** yellow powder; molecular formula C_26_H_28_O_4_; melting point = 181.8 °C. ^13^C NMR (CD_3_OD, 150 MHz) *δ*_*C*_: 166.6 (C-2), 104.5 (C-3), 184.0 (C-4), 162.9 (C-5), 100.9 (C-6), 164.4 (C-7), 95.9 (C-8), 158.9 (C-9), 107.0 (C-10), 123.4 (C-1′), 110.4 (C-2′), 149.5 (C-3′), 152.3 (C-4′), 116.7 (C-5′), 121.9 (C-6′), 56.6 (C-7′) for aglycone; 100.6 (C-1′′), 78.6 (C-2′′), 77.9 (C-3′′), 70.9 (C-4′′), 66.9 (C-5′′), 110.0 (C-1′′′), 78.1 (C-2′′′), 80.7 (C-3′′′), 65.8 (C-4′′′), 75.4 (C-5′′′) for sugar moiety. ^1^H NMR data (CD_3_OD, 600 MHz) *δ*_*H*_: 6.70 (1H, s, H-3), 6.45 (1H, d, *J* = 2.1 Hz, H-6), 6.77 (1H, d, *J* = 2.1 Hz, H-8), 7.52 (1H, d, *J* = 2.1 Hz, H-2′), 6.95 (1H, d, *J* = 8.4 Hz, H-5′), 7.56(1H, dd, *J* = 8.4 and 2.1 Hz, H-6′), 3.98 (3H, s, H-7′) for aglycone; 5.16 (1H, d, *J* = 7.1 Hz, H-1′′), 3.68 (1H, dd, *J* = 9.0 and 7.1, H-2′′), 3.63 (1H, m, H-3′′), 3.62 (1H, m, H-4′′), 3.98 (1H, m, H-5′′a), 3.48 (1H, t, *J* = 9.6, H-5′′b), 5.46 (1H, d, *J* = 1.7 Hz, H-1′′′), 3.98 (1H, m, H-2′′′), 3.56 (2H, brs, H-4′′′), 4.05 (1H, d, *J* = 9.4, H-5′′′a), 3.84 (1H, d, *J* = 9.4, H-5′′′b) for sugar moiety.

Chrysoeriol-7-*O*-*α*-L-rhamnopyranosyl-(1 → 6)-*β*-D-(4′′-hydrogenosulfate) glucopyranoside (**4):** yellow amorphous powder; molecular formula C_28_H_31_NaO_18_S. ^13^C NMR data (CD_3_OD, 150 MHz) *δ*_*C*_: 166.8 (C-2), 104.5 (C-3), 184.1 (C-4), 163.1 (C-5), 101.1 (C-6), 164.0 (C-7), 96.2 (C-8), 159.0 (C-9), 107.2 (C-10), 123.6 (C-1′), 110.8 (C-2′), 149.6 (C-3′), 152.3 (C-4′), 116.9 (C-5′), 122.0 (C-6′), 56.7 (C-7′) for aglycone; 100.9 (C-1′′), 74.5 (C-2′′), 76.8 (C-3′′), 77.5 (C-4′′), 75.3 (C-5′′), 67.1 (C-6′′), 102.4 (C-1′′′), 71.9 (C-2′′′), 72.3 (C-3′′′), 74.2 (C-4′′′), 69.8 (C-5′′′), 17.9 (C-6′′′) for sugar moiety. ^1^H NMR data (CD_3_OD, 600 MHz) *δ*_*H*_: 6.73 (1H, s, H-3), 6.56 (1H, d, *J* = 2.1 Hz, H-6), 6.84 (1H, d, *J* = 2.1 Hz, H-8), 7.55 (1H, d, *J* = 2.1 Hz, H-2′), 6.98 (1H, d, *J* = 8.4 Hz, H-5′), 7.59 (1H, dd, *J* = 8.4 and 2.1 Hz, H-6′), 3.99 (3H, s, H-7′) for aglycone; 5.15 (1H, d, *J* = 7.8 Hz, H-1′′), 3.61 (1H, dd, *J* = 9.1 and 7.8 Hz, H-2′′), 3.84 (1H, t, *J* = 9.1 Hz, H-3′′), 4.32 (1H, dd, *J* = 9.9 and 9.1 Hz, H-4′′), 3.89 (1H, m, H-5′′), 4.10 (1H, m, H-6′′a), 3.68 (1H, m, H-6′′b), 4.75 (1H, d, *J* = 1.3 Hz, H-1′′′), 3.95 (1H, dd, *J* = 3.4 and 1.3, H-2′′′), 3.72 (1H, dd, *J* = 9.5 and 3.4 Hz, H-3′′′), 3.32 (1H, t, *J* = 9.5 Hz, H-4′′′), 3.62 (1H, m, H-5′′′), 1.21 (3H, d, *J* = 6.2 Hz, H-6′′′) for sugar moiety.

Isorhamnetin-3-*O*-*α*-L-rhamnopyranosyl-(1 → 6)-*β*-D-glucopyranoside (**5):** yellow amorphous powder; molecular formula C_28_H_32_O_15_. ^13^C NMR data (CD_3_OD, 150 MHz) *δ*_*C*_: 159.8 (C-2), 135.7 (C-3), 179.8 (C-4), 162.4 (C-5), 104.3 (C-6), 160.1 (C-7), 100.2 (C-8), 157.5 (C-9), 108.6 (C-10), 123.0 (C-1′), 114.5 (C-2′), 148.4 (C-3′), 151.1 (C-4′), 116.2 (C-5′), 124.4 (C-6′), 56.7 (C-7′) for aglycone; 104.0 (C-1′′), 75.9 (C-2′′), 78.1 (C-3′′), 71.8 (C-4′′), 77.4 (C-5′′), 68.7 (C-6′′), 102.6 (C-1′′′), 72.1 (C-2′′′), 72.3 (C-3′′′), 73.8 (C-4′′′), 69.8 (C-5′′′), 18.0 (C-6′′′) for sugar moiety. ^1^H NMR data (CD_3_OD, 600 MHz) *δ*_*H*_: 6.71 (1H, d, *J* = 2.2 Hz, H-6), 7.09 (1H, d, *J* = 2.2 Hz, H-8), 7.91 (1H, d, *J* = 2.2 Hz, H-2′), 6.95 (1H, d, *J* = 8.5 Hz, H-5′), 7.72 (1H, dd, *J* = 8.5 and 2.2 Hz, H-6′), 3.98 (3H, s, H-7′) for aglycone; 5.30 (1H, d, *J* = 7.6 Hz, H-1′′), 3.49 (1H, dd, *J* = 9.1 and 7.6 Hz, H-2′′), 3.45 (1H, t, *J* = 9.1 Hz, H-3′′), 3.23 (1H, t, *J* = 9.1 Hz, H-4′′), 4.40 (1H, m, H-5′′), 3.84 (1H, dd, *J* = 12.1 and 1.9 Hz, H-6′′a), 3.42 (1H, m, H-6′′b), 4.53 (1H, d, *J* = 1.3 Hz, H-1′′′), 3.58 (1H, dd, *J* = 3.4 and 1.3, H-2′′′), 3.47 (1H, dd, *J* = 9.5 and 3.4 Hz, H-3′′′), 3.25 (1H, t, *J* = 9.5 Hz, H-4′′′), 3.41 (1H, m, H-5′′′), 1.10 (3H, d, *J* = 6.2 Hz, H-6′′′) for sugar moiety.

### Antimicrobial assay

#### Microorganisms

The microorganisms used in this study were consisted of five bacterial strains namely *Staphylococcus aureus* ATCC 25923*, Vibrio cholerae* NB2, PC2, SG24 (1) and CO6 [16]. Also included were two fungi *Candida albicans* ATCC 9002 and *Cryptococcus neoformans* IP95026. These bacteria and yeasts were obtained from our local stocks.

#### Determination of minimum inhibitory concentration and minimum microbicidal concentration

The minimum inhibitory concentration **(**MIC) values were determined using the broth micro-dilution method as described earlier [17]. The MIC values were defined as the lowest sample concentration that prevented the change in color indicating a complete inhibition of microbial growth. The lowest concentrations that yielded no growth after the subculturing were taken as the minimum microbicidal concentration (MMC) values [18]. Ciprofloxacin (Sigma-Aldrich, Steinheim, Germany) and amphotericin B (Merck, Darmstadt, Germany) were used as positive controls for bacteria and yeast respectively.

### Study on mode of action

#### MIC and MBC changes under osmotic stress condition

Osmotic stress was induced by adding 5% NaCl (*w*/*v*) to MHB. The MHB supplemented with 5% NaCl was then sterilized and used for the determination of a new MIC and MBC values of the samples as previously described [17]. The incubation time was increased from 24 to 48 h at 37 °C.

#### Effect of isolated compounds on cell membrane

The alteration of cell membrane of *V. cholerae* NB2 was evaluated by measuring the optical densities at 260 nm of the bacterial suspensions in the presence and absence of compounds **1–5** using the method described by Carson et al. [19]. For this purpose, the compounds were tested at their MIC using 1 mL of the bacterial suspension (approximately 10^8^ CFU/mL). The mixture was then incubated at 37 °C at different time intervals (0: immediately after addition of the compound; 15; 30; 60 min), 50 μL of the mixture was taken and mixed with 1.95 mL of Phosphate Buffered Saline (PBS Buffer). The absorbance was measured on the spectrophotometer at 260 nm against the blank (PBS). For the negative control, 1 mL of bacterial suspension was incubated at 37 °C and 50 μL of the suspension was removed at the end of the various incubation times and mixed with 1.95 mL of Buffer. The optical densities were read in the same way.

#### Bacteriolytic assay

The bacteriolytic activities of the isolated compounds were determined using the time-kill kinetic method as previously described [20] with slight modifications. Full growth of *V. cholerae* NB2 in MHB was diluted 100 times and incubated at 37 °C to produce an OD_600_ of 0.8 as starting inoculum. Sample solutions were added to the starting bacterial suspension to give a final concentration of 2 × MIC and incubated at 37 °C with shaking, then 100 μL was removed from each tube at 0, 15, 30, 60, and 120 min and the optical density measured at 600 nm. Vancomycin and tetracycline were used as positive controls and the tubes without isolated compounds served as negative controls.

#### Hemolytic assay

Whole blood (10 mL) from albino rats was collected by cardiac puncture in EDTA tubes. The study was conducted according to the ethical guidelines of the Committee for Control and Supervision of Experiments on Animals (Registration no. 173/CPCSEA, dated 28 January, 2000), Government of India, on the use of animals for scientific research. Erythrocytes were harvested by centrifugation at room temperature for 10 min at 1000 x *g* and were washed three times in PBS buffer [21]. The cytotoxicity was evaluated as previously described [21].

### Statistical analysis

Data were analyzed by one-way analysis of variance followed by Waller-Duncan post hoc test. The experimental results were expressed as the mean ± Standard Deviation (SD). Differences between groups were considered significant when *p* < 0.05. All analyses were performed using the Statistical Package for Social Sciences (SPSS, version 12.0) software.

## Results

### Chemical analysis

**Fig. 1:**
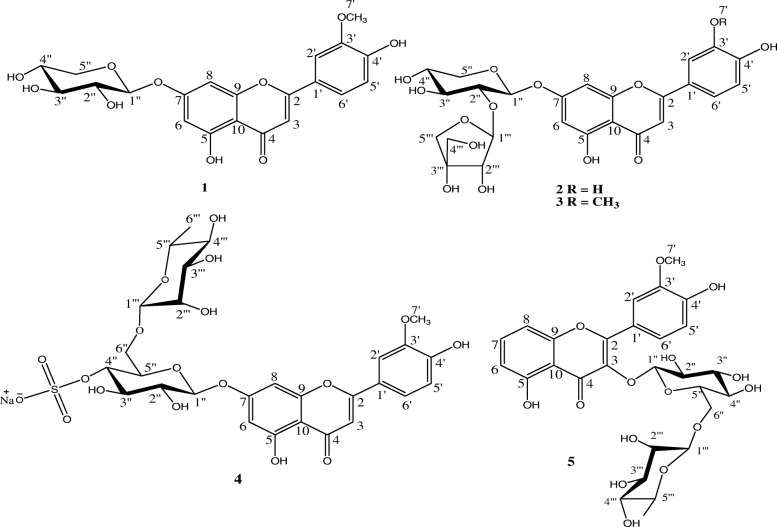
Chemical structures of flavonoids (**1–5**) isolated from *n*-BuOH extract of aerial parts of *G. grandulosum* Turill. **1**:chrysoeriol-7-*O*-*β*-D-xyloside; **2:**luteolin-7-*O*-*β*-D-apiofuranosyl-(1 → 2)-*β*-D-xylopyranoside; **3**: chrysoeriol-7-*O*-*β*-D-apiofuranosyl-(1 → 2)-*β*-D-xylopyranoside; **4**: chrysoeriol-7-*O*-*α*-L-rhamnopyranosyl-(1 → 6)-*β*-D-(4"-hydrogenosulfate) glucopyranoside**; 5:**Isorhamnetin-3-*O*-*α*-L-rhamnopyranosyl-(1 → 6)-*β*-D-glucopyranoside

The structures of five known flavonoid glycosides isolated from the *n*-BuOH fraction of leaves of *G. grandulosum* (Fig. 1) were determined using spectroscopic analysis and NMR spectra in conjunction with 2D experiments (COSY, NOESY, HSQC and HMBC). Direct comparison with published information led to the identification of chrysoeriol-7-*O*-*β*-D-xyloside **1** [22], luteolin-7-*O*-*β*-D-apiofuranosyl-(1 → 2)-*β*-D-xylopyranoside **2** [23], chrysoeriol-7-*O*-*β*-D-apiofuranosyl-(1 → 2)-*β*-D-xylopyranoside **3** [13], chrysoeriol-7-*O*-*α*-L-rhamnopyranosyl-(1 → 6)-*β*-D-(4′′-hydrogeno sulfate) glucopyranoside **4** [13] and isorhamnetin-3-*O*-*α*-L-rhamnopyranosyl-(1 → 6)-*β*-D-glucopyranoside **5** [24].

### Antimicrobial activity

The in vitro activities of MeOH, *n*-BuOH and EtOAc extracts as well as their isolated compounds against pathogenic bacteria and fungi are presented in Table 1. The test samples demonstrated varying degrees of inhibitory activities against the bacterial and fungal strains. Fungal strains were generally more susceptible to the effects of the compounds, but less susceptible to extracts. The EtOAc and *n*-BuOH extracts were active against *C. albicans* and *C. neoformans* which were not susceptible to the MeOH extract. The MIC values obtained with the EtOAc and *n*-BuOH extracts were smaller than those obtained with the MeOH extract. These observations suggest that the fractionation of the MeOH extract enhanced its antimicrobial activity. The lowest MIC values were recorded on *S. aureus*; suggesting that this microorganism was the most susceptible to all the test samples. The EtOAc extract showed the highest antimicrobial activity when compared with the MeOH and *n*-BuOH extracts.

**Table 1:** Antimicrobial activities of extracts, isolated compounds and reference antimicrobial drugs

Extracts/Compounds	Inhibition parameters	*V. cholerae* SG24 (1)	*V. cholerae* CO6	*V. cholerae* NB2	*V. cholerae* PC2	*S. aureus* ATCC 25923	*C. albicans* ATCC 9002	*C. neoformans* IP95026
MeOH extract	MIC	512	512	256	512	256	> 2048	> 2048
	MMC	512	512	512	1024	512	/	/
	MMC/MIC	1	1	2	2	2	/	/
*n*-BuOH extract	MIC	256	256	128	128	128	2048	2048
	MMC	256	256	128	256	128	> 2048	> 2048
	MMC/MIC	1	1	1	2	1	/	/
EtOAc extract	MIC	64	128	64	64	64	1024	512
	MMC	128	128	64	64	64	1024	1024
	MMC/MIC	2	1	1	1	1	1	2
1	MIC	16	8	8	8	4	32	8
	MMC	16	8	16	8	8	64	8
	MMC/MIC	1	1	2	1	2	2	1
2	MIC	16	16	8	8	4	64	16
	MMC	32	16	16	8	8	64	32
	MMC/MIC	2	1	2	1	2	1	2
3	MIC	32	64	32	32	8	32	16
	MMC	64	128	32	64	16	32	16
	MMC/MIC	2	2	1	2	2	1	1
4	MIC	8	8	8	8	4	8	4
	MMC	16	8	8	8	4	8	8
	MMC/MIC	2	1	1	1	1	1	2
5	MIC	32	16	16	16	8	64	32
	MMC	32	16	16	16	8	128	64
	MMC/MIC	1	1	1	1	1	2	2
Ref^a^	MIC	32	4	16	16	0.5	0.5	0.25
	MBC	32	4	16	16	0.5	0.5	0.25
	MBC/MIC	1	1	1	1	1	1	1

/: not determined; *MIC* Minimum Inhibitory Concentration, *MMC* Minimum Microbicidal Concentration; the MIC and MMC were measured in μg/mL; ^a^: amphotericin B for yeasts and ciprofloxacin for bacteria

The antimicrobial activities of the isolated compounds from *G. glandulosum* were as follows: compound **4** > compound **1** > compound **2** > compound **5** > compound **3**. The lowest MIC value of 4 μg/mL was recorded on *C. neoformans* with compound **4** and on *S. aureus* with compounds **1**, **2** and **4** whereas the lowest MMC value was obtained on *S. aureus* with compound **4.** However, the highest MIC value for compounds (64 μg/mL) was recorded with compound **3** against *V. cholerae* CO6, and with compounds **2** and **5** against *C. albicans*, while the highest MBC value of 128 μg/mL was obtained on *V. cholerae* CO6 with compound **3** and on *C. albicans* with compound **5**.

### Antibacterial activity of flavonoid glycosides under osmotic stress condition

The MIC values of flavonoid glycosides against Gram-negative and Gram-positive bacteria are reported in Table 2. The results clearly showed that the MIC values of flavonoid glycosides obtained under osmotic stress (in the presence of 5% NaCl) are smaller than those obtained under normal conditions (0% NaCl). This result suggests an increase in the activity of purified flavonoid glycosides under osmotic stress. As demonstrated under normal condition, compound **4** was still the most effective under osmotic stress, followed in decreasing order by compounds **1** and **2**. The MIC values of chloramphenicol determined under osmotic stress condition were smaller than those determined under normal conditions. However, all the MIC values of vancomycin determined under osmotic stress were higher than those determined under normal conditions. Table 1 further shows that under osmotic stress, the antibacterial activities of compounds **1**, **2** and **4** against *V. chorae* SG24 (1), *V. chorae* CO6, *V. chorae* NB2 and *V. chorae* PC2 were higher than that of vancomycin.

**Table 2:** Antibacterial activities in terms of MIC (μg/mL) of compounds 1, 3 and 4 under osmotic stress condition against bacterial strains

Bacteria	Compound 1		Compound 2		Compound 4		Chloramphenicol		Vancomycin	
	0% NaCl	5% NaCl	0% NaCl	5% NaCl	0% NaCl	5% NaCl	0% NaCl	5% NaCl	0% NaCl	5% NaCl
*V. chorae* SG24 (1)	16	8	16	16	8	4	4	1	16	64
*V. cholerae CO6*	8	4	16	4	8	2	16	2	16	32
*V. cholerae* NB2	8	4	8	2	8	2	64	1	32	64
*V. cholerae* PC2	8	4	8	2	8	2	16	1	32	64
*S. aureus*	4	2	4	2	4	1	32	0.5	0,5	1

### Effect of flavonoid glycosides on cell membrane

**Fig. 2:**
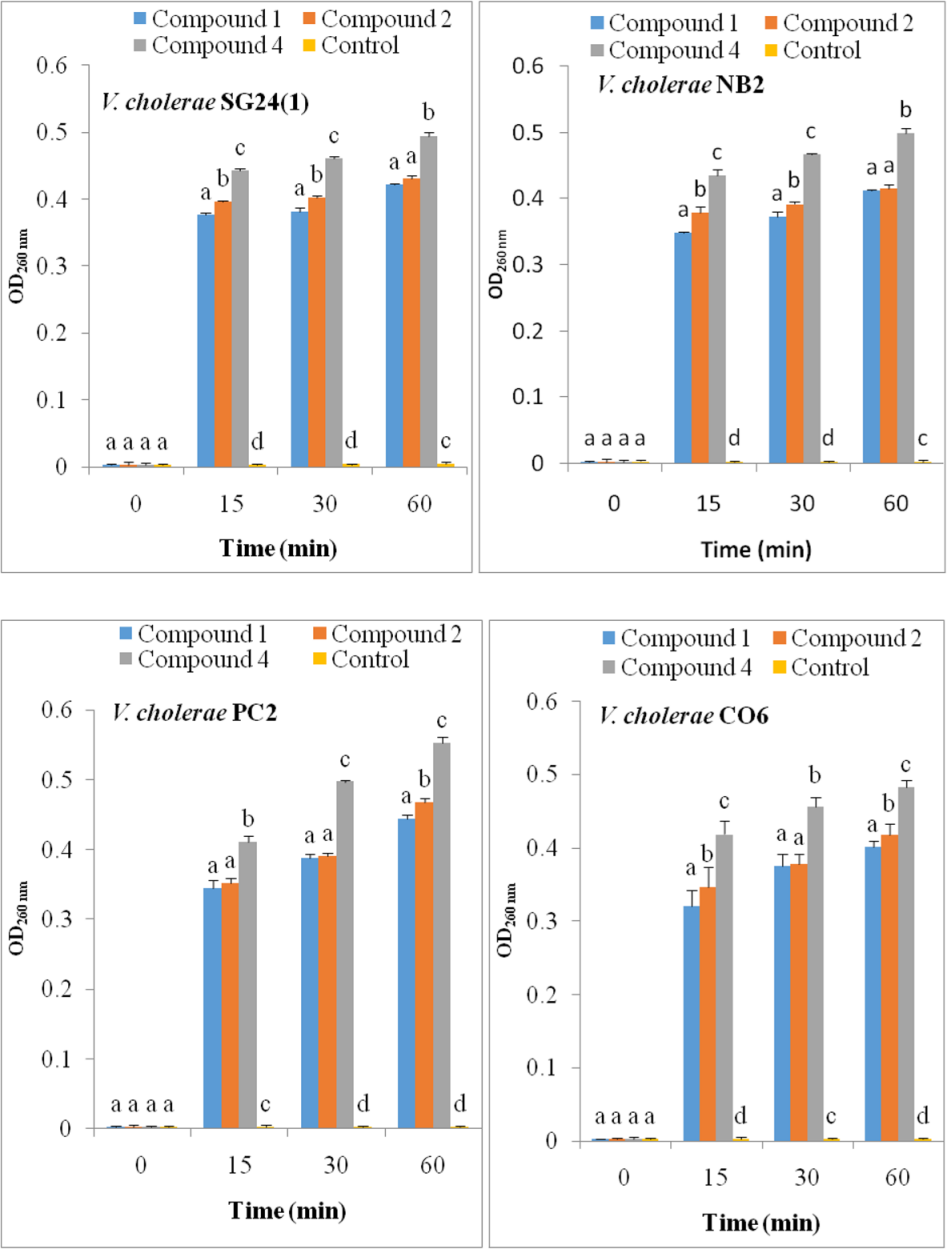
Appearance of 260-nm-absorbing material in the filtrates of *V. cholerae* SG24 (1), PC2, NB2 and CO6 after treatment with compounds **1, 2** and **4**. Bars represent the mean ± standard deviation of the triplicate OD at each incubation time. At the same incubation time, letters a-d indicate significant differences between samples according to one way ANOVA and Waller Duncan test; *p* < 0.05

The effect of flavonoid glycosides of *G. glandulosum* was evaluated in terms of leakage of UV 260 absorbing material through the bacterial cell membrane (Fig. 2). After treatment with flavonoid glycosides at MIC values of compounds **1**, **2** and **4**, the OD_260_ values of filtrates of all test strains increased and most of the leakage occurred during the initial period (≤ 15 min), followed by a slight increase with prolonged incubation period. At the same time, the OD_260_ of the control without compound was not changed. These results suggest that flavonoid glycosides from *G. glandulosum* damage the cytoplasmic membrane and cause loss of intracellular components. The highest values of OD_260_ were recorded with compound **4** for all the *V. cholerae* strains, whereas the least OD_260_ values were noticed with compound **2**, indicating that compound **4** released the highest amounts of nucleic acids followed in decreasing order by compound **1**, then **2**.

### Bacteriolytic effect of compounds 1, 2 and 4

**Fig. 3:**
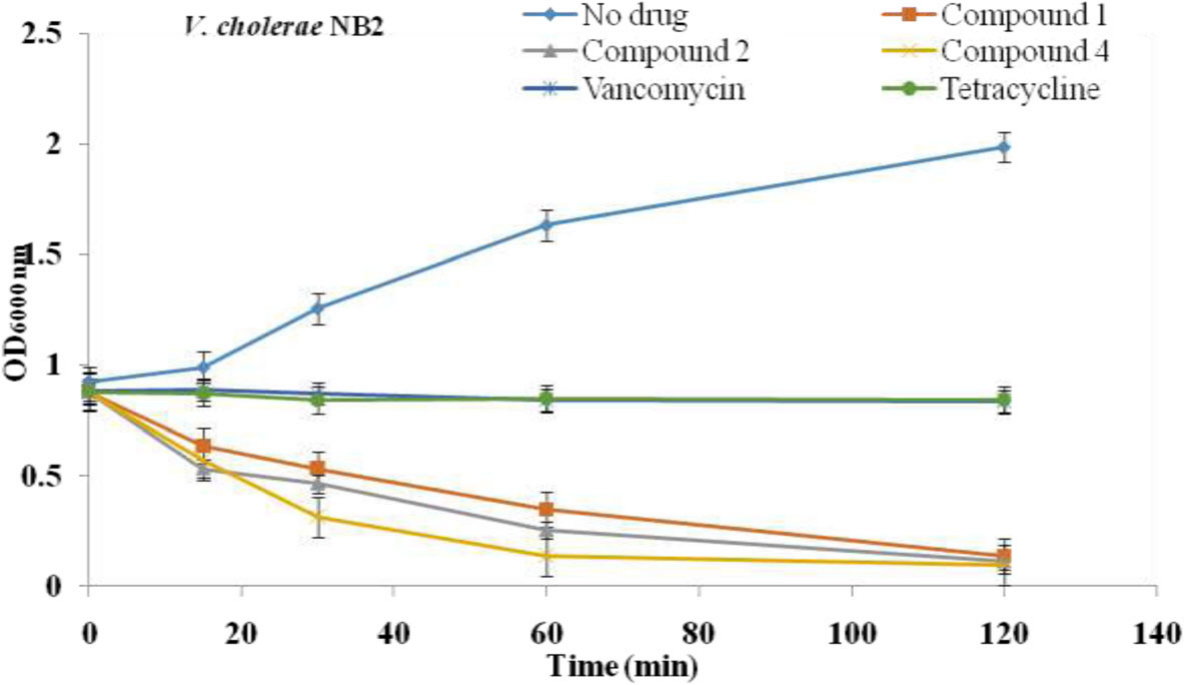
Bacteriolytic effect of compounds **1**, **2** and **4** against *V. cholerae* NB2. Results represent the mean ± standard deviation of the triplicate OD at each incubation time

The result on the leakage of 260 nm absorbing material was consistent with that of bacteriolysis (Fig. 3). This result showed a decrease in the optical density of suspension treated with compounds **1**, **2** and **4**. After 120 min, compounds **1**, **2** and **4** induced a decline in cell turbidity of 93.20, 94.36 and 95.16%, respectively in bacteria suspension compared to time 0, indicating the lysis of bacterial cells.

### Haemolytic activity

The haemolytic activities of extracts and compounds **1–5** against red blood cells (RBCs) were investigated using Triton X-100 as a positive control. The positive control showed about 100% lysis, whereas the phosphate buffer saline (PBS) showed no lysis of RBCs. Interestingly, none of the tested samples showed haemolytic activities against RBCs at concentrations up to 256 and 2048 μg/mL for isolated compounds and extracts respectively (results not shown). This finding highlights the fact that the observed biological efficacy was not due to haemolysis.

## Discussion

The antimicrobial activity of a plant extract is considered to be highly active if the MIC < 100 μg/mL; significantly active when 100 ≤ MIC ≤512 μg/mL; moderately active when 512 < MIC ≤2048 μg/mL; weakly active if MIC > 2048 μg/mL and not active when MIC > 10,000 μg/mL [25]. Hence, the EtOAc extract of *G. glandulosum* was highly active (MIC < 100 μg/mL) against *V. cholerae* SG24 (1), *V. cholerae* NB2, *V. cholerae* PC2 and *S. aureus*; significantly active (100 ≤ MIC ≤512 μg/mL) against *V. cholerae* CO6 and *C. neoformans*; moderately active (512 < MIC ≤2048 μg/mL) on *C. albicans*. The MeOH and *n*-BuOH extracts were significantly active against the test bacterial species; weakly and moderately active against the yeast cells respectively.

In this study, we also investigated if the mode of action of flavonoid compounds is bactericidal or bacteriostatic. The results of the MMC values were fourfold lesser than their corresponding MIC values. This observation suggests that the actions of extracts from *G. glandulosum* and their isolated flavonoid glycosides were bactericidal [11].

The antibacterial activities of flavonoid glycosides were in some cases equal to, or higher than those of ciprofloxacin used as reference antibiotic, suggesting that they might be effective antibiotics against these pathogenic bacteria. Taking into account the medical importance of the test microbial species, the result can be considered as promising for the development of new antimicrobial drugs. The antimicrobial activities of purified flavonoids corroborated with those of early reports against bacteria and fungi [5, 11, 26–28]. The antibacterial activity of the samples against *V. cholerae* and *S. flexneri* are particularly noteworthy since these strains were MDR clinical isolates which were resistant to commonly used drugs such as ampicillin, streptomycin, nalidixic acid, furazolidone and co-trimoxazole [16, 29, 30].

Antimicrobial cutoff points have been defined in the literature to enable the understanding of the effectiveness of pure compounds as follows: highly active: MIC below 1 μg/mL (or 2.5 μM), significantly active: 1 ≤ MIC ≤10 μg/mL (or 2.5 ≤ MIC < 25 μM), moderately active: 10 < MIC ≤100 μg/mL (or 25 < MIC ≤250 μM), weakly active: 100 < MIC ≤1000 μg/mL (or 250 < MIC ≤2500 μM and not active: MIC > 1000 μg/mL (or > 2500 μM) [25]. Based on this, the antimicrobial activities of all the tested flavonoid glycosides could be considered as significant or moderate against the specific microorganisms.

The antimicrobial activities of the isolated compounds from *G. glandulosum* were in this order: compound **4** > compound **1** > compound **2** > compound **5** > compound **3**. Very little is known about the structure–function relationships of natural antimicrobials, but it seems that different substituent groups within the compounds had a great influence on their biophysical and biological properties [31]. Structural features such as the presence of an aromatic ring, the sugar moiety or the numbers of hydroxyl and methoxyl groups can significantly change membrane permeability and subsequent affinity to external and internal binding sites in the bacteria, thus influencing the compound’s antimicrobial properties [32].

The antibacterial activities of flavonoid glycosides and chloramphenicol increased under osmotic stress (5% NaCl) whereas that of vancomycin decreased under this condition. The results were supported by the observation that certain bacterial strains (*E. coli, S. aureus, P. aeruginosa*) can survive under osmotic stress conditions [33]. At low water activity, lipid composition of bacterial cell membrane was changed [34]. This incident might lead to occurrence of more antibacterial binding site on cell membrane of bacteria and cause less resistance to antibacterial substance. Therefore, the presence of the salt triggered changes in the membrane lipid composition. This is possible to increase the antibacterial activity of flavonoid glycosides and chloramphenicol. However, the mechanisms that make bacteria more sensitive to certain antibiotics under osmotic stress conditions are still unknown. The results of vancomycin activity are in agreement with those of McMahon and coworkers [35] who demonstrated a decrease in the activity of amikacin, ceftriaxone and trimethoprim against *E. coli* and *S. aureus* under osmotic stress conditions.

Marked leakage of cytoplasmic material is considered indicative of gross and irreversible damaged to the cytoplasmic membrane. Many antibacterial compounds that act on the bacterial cytoplasmic membrane induce the loss of 260 nm-absorbing materials (nucleic acids) including chlorohexidine, hexachlorophene, phenetyl alcohol, tetracycline, polymixin, α-pinene, and lemon grass oil [19]. The *V. cholerae* suspension treated with flavonoid glycosides, showed a significant increase in the optical density at 260 nm, suggesting that nucleic acids were lost through a damaged cytoplasmic membrane.

Our observations confirm that the antimicrobial activity of flavonoid glycosides results from their ability to disrupt the permeability barrier of microbial membrane structures. This mode of action is similar to that of other broad-spectrum, membrane-active disinfectants and preservatives, such as phenol derivatives, chlorohexidine and para benzoic acid derivatives [36]. Furthermore, Devi and Kapila [37], reported the antibacterial mechanism as disruption of plasma membrane by the phytochemicals in the extracts of Indian liverworts.

The fact that flavonoids-induced damage to cell membrane structure accompanied by the decline in the absorbance of bacterial cell suspension treated with compounds has confirmed it as the most likely cause of cell death. Our result is supported by the observation that other flavonoid compounds such as epigallocatechin gallate and galangin induced 3-log reduction or more in viable counts of *S. aureus* [38, 39].

## Conclusions

The results of the present study indicate that the purified flavonoid glycosides from *G. glandulosum* possess antimicrobial activities. Their mode of antibacterial activity is due to cell lysis and disruption of the cytoplasmic membrane by action upon the membrane permeability leading to leakage of cellular components and eventually cell death. This will lead to improve antimicrobial formulations and to ensure the prevention of the emergence of microbial resistance. However, the possibility remains that sites of action other than the cytoplasmic membrane exist. Further work is required to expatiate fully the mechanisms involved.

## Abbreviations

^13^C-NMR: Carbon Thirteen Nuclear Magnetic Resonance
^1^H NMR: Proton Nuclear Magnetic Resonance
^2^D NMR: Two-dimension Nuclear Magnetic Resonance
ATCC: American Type Culture Collection
CC: Column Chromatography
COSY: Correlation Spectroscopy
DMSO: Dimethylsulfoxide
EtOAc: Ethyl acetate
HMBC: Heteronuclear Multiple Bond Connectivities
HNC: *Herbier National du Cameroun*
HR-EI-MS: High Resolution Electron Impact Mass Spectrometry
HR-TOFESIMS: High-Resolution Time of Flight Electrospray Ionization Mass Spectrometry
HSQC: The Heteronuclear Single Quantum Coherence
IR: Infra-red
MBC: Minimum bactericidal concentration
MDR: Multi-Drug-Resistant
MeOH: Methanol
MHA: Mueller Hinton agar
MHB: Mueller Hinton broth
MIC: Minimum inhibitory concentration
MMC: Minimum Microbicidal Concentration
NA: Nutrient agar
n-BuOH: n-Butanol
NMR: Nuclear Magnetic Resonance
Rf: Retention factor
TLC: Thin Layer Chromatography
TMS: Tetramethylsilane
TOF-ESIMS: Time of Flight Electrospray Ionization Mass Spectrometry
UV: Ultra-violet

## References

[1] Nair GB, Ramamurthy T, Bhattacharya MK, Krishnan T, Ganguly S, Saha DR (2010). Emerging trends in the etiology of enteric pathogens as evidenced from an active surveillance of hospitalized diarrhoeal patients in Kolkata India. Gut Pathog.

[2] Mahady GB, Huang Y, Doyle BJ, Locklear T (2008). Natural products as antibacterial agents. Nat Prod Chem.

[3] Tatsimo NSJ, Tamokou JD, Lamshöft M, Mouafo TF, Lannang MA, Sarkar P (2015). LC-MS guided isolation of antibacterial and cytotoxic constituents from *Clausena anisata*. Med Chem Res.

[4] Pagning NAL, Tamokou JD, Lateef M, Tapondjou AL, Kuiate JR, Ngnokam D (2016). New triterpene and new flavone glucoside from *Rhynchospora corymbosa* (Cyperaceae) with their antimicrobial tyrosinase and butyrylcholinesterase inhibitory activities. Phytochem Lett.

[5] Tebou PLF, Tamokou JD, Ngnokam D, Voutquenne-Nazabadioko L, Kuiate JR, Bag PK (2017). Flavonoids from *Maytenus buchananii* as potential cholera chemotherapeutic agents. S Afr J Bot.

[6] Robinson T (1991). The organic constituents of higher plants – their chemistry and interrelationships.

[7] Mills S, Bone K. Principles and practice of phytotherapy –modern herbal medicine. New York: Churchill Livingstone. 2000:31–4.

[8] Evans WC (2002). Trease and Evans Pharmacognosy.

[9] Benavente-Garcia O, Castillo J, Marin FR, Ortuno A, Del Rio JA (1997). Uses and properties of citrus flavonoids. J Agric Food Chem.

[10] Vuorela S, Kreander K, Karonen M, Nieminen R, Hämäläinen M, Galkin A (2005). Preclinical evaluation of rapeseed raspberry and pine bark phenolics for health related effects. J Agric Food Chem.

[11] Djouossi MG, Tamokou JD, Ngnokam D, Kuiate JR, Tapondjou AL, Harakat D (2015). Antimicrobial and antioxidant flavonoids from the leaves of *Oncoba spinosa* Forssk (Salicaceae). BMC Complement Altern Med.

[12] Barker RM (1986). Graptophyllum nees. J Adel Bot Gard.

[13] Ngoufack TC, Ngnokam D, Harakat D, Voutquenne-Nazabadioko L (2017). Three new flavonoid glycosides from the aerial parts of *Graptophyllum grandulosum* Turril (Acanthaceae). Phytochem Lett.

[14] Wahyuningtyas E (2005). The *Graptophylum pictum* effect on acrylic resin complete denture plaque growth. Dent J (Maj Kedokt Gigi).

[15] Jiangseubchatveera N, Liawruangrath B, Liawruangrath S, Teerawutgulrag A, Santiarwarn D, Korth J (2015). The chemical constituents and the cytotoxicity antioxidant and antibacterial activities of the essential oil of *Graptophyllum pictum* (L) Griff. J Essent Oil Bear Pl.

[16] Bag PK, Bhowmik P, Hajra TK, Ramamurthy T, Sarkar P, Majumder M (2008). Putative virulence traits and pathogenicity of *Vibrio cholerae* non-O1 non-O139 isolated from surface waters in Kolkata India. Appl Environ Microbiol.

[17] Fondjo ES, Dimo KSD, Tamokou JD, Tsemeugne J, Kouamo S, Ngouanet D (2016). Synthesis characterization antimicrobial and antioxidant activities of the homocyclotrimer of 4-Oxo-4h-thieno[34-C]chromene-3diazonium sulfate. Open Med Chem J.

[18] Joubouhi C, Tamokou JD, Ngnokam D, Voutquenne-Nazabadioko L, Kuiate JR (2017). Iridoids from *Canthium subcordatum* iso-butanol fraction with potent biological activities. BMC Complement Altern Med.

[19] Carson CF, Mee BJ, Riley TV (2002). Mechanism of action of *Melaleuca alternifolia* (tea tree) oil on *Staphylococcus aureus* determined by time kill, lysis, leakage and salt tolerance assays and electron microscopy. Antimicrob Agents Chemother.

[20] Ooi N, Miller K, Hobbs J, Rhys-Williams W, Love W, Chopra I (2009). XF-73 a novel antistaphylococcal membrane active agent with rapid bactericidal activity. J Antimicrob Chemother.

[21] Situ H, Bobek LA (2000). *In vitro* assessment of antifungal therapeutic potential of salivary histatin-5 two variants of histatin-5 and salivary mucin (MUC7) domain 1. Antimicrob Agents Chemother.

[22] Markham KR, Ternai B, Stanley R, Geiger H, Mabry TJ (1978). Carbon-13 NMR studies of flavonoids III naturally occurring flavonoid glycosides and their acylated derivatives. Tetrahedron.

[23] Koffi EN, Le Guernevéc C, Lozanoa PR, MeudeccAdjéd FA, Bekrob YA, Lozanoa YF (2013). Polyphenol extraction and characterization of *Justicia secunda* Vahl leaves for traditional medicinal uses. Ind Crop Prod.

[24] Mona-Antonia B, Hanns H (1999). Flavonol glycosides from *Eschscholtzia californica*. Phytochemistry.

[25] Tamokou J.D.D., Mbaveng A.T., Kuete V. (2017). Antimicrobial Activities of African Medicinal Spices and Vegetables. Medicinal Spices and Vegetables from Africa.

[26] Tatsimo NSJ, Tamokou JD, Havyarimana L, Dezső C, Forgo P, Hohmann J (2012). Antimicrobial and antioxidant activity of kaempferol rhamnoside derivatives from *Bryophyllum pinnatum*. BMC Res Notes.

[27] Mabou FD, Tamokou JD, Ngnokam D, Voutquenne-Nazabadioko L, Kuiate JR, Bag PK (2016). Complex secondary metabolites from *Ludwigia leptocarpa* with potent antibacterial and antioxidant activities. Drug Discover Therap.

[28] Tatsimo NSJ, Tamokou JD, Tsague TV, Lamshoft M, Sarkar P, Bag PK (2017). Antibacterial-guided isolation of constituents from *Senna alata* leaves with a particular reference against multi-drug-resistant *Vibrio cholerae* and *Shigella flexneri*. Int J Biol Chem Sci.

[29] Thakurta P, Bhowmik P, Mukherjee S, Hajra TK, Patra A, Bag PK (2007). Antibacterial antisecretory and antihemorrhagic activity of *Azadirachta indica* used to treat cholera and diarrhea in India. J Ethnopharmacol.

[30] Acharyya S, Sarkar P, Saha DR, Patra A, Ramamurthy T, Bag PK (2015). Intracellular and membrane damaging activities of methyl gallate isolated from *Terminalia chebula* against multi-drug resistant *Shigella* species. J Med Microbiol.

[31] Mandalari G, Bennett RN, Bisignano G, Trombetta D, Saija A, Faulds CB (2007). Antimicrobial activity of flavonoids extracted from bergamot (*Citrus bergamia* Risso) peel a byproduct of the essential oil industry. J Appl Microbiol.

[32] Fitzgerald DJ, Stratford M, Gasson MJ, Ueckert J, Bos A, Narbad A (2004). Mode of antimicrobial action of vanillin against *Escherichia coli, Lactobacillus plantarum* and *Listeria innocua*. J Appl Microbiol.

[33] Besten HMW, Mols M, Moezelaar R, Zwietering MH, Abee T (2009). Phenotypic and transcriptomic analyses of mildly and severely salt-stressed *Bacillus cereus* ATCC 14579 cells. Appl Environ Microbiol.

[34] Beales N (2004). Adaptation of microorganisms to cold temperatures weak acid reservatives low pH and osmotic stress: a review. Compr Rev Food Sci Food Saf.

[35] McMahon MAS, Xu J, Moore JE, Blair IS, McDowell DA (2007). Environmental stress and antibiotic resistance in food-related pathogens. Appl Environ Microbiol.

[36] Cox SD, Mann CM, Markham JL, Bell HC, Gustafson JE, Warmington JR (2000). The mode of antibacterial action of the essential oil of *Melaleuca alternifolia* (tea tree oil). J Appl Microbiol.

[37] Devi K, Kapila S (2013). Antibacterial effect of some Indian liverworts. Int J Pharm Sci Rev Res.

[38] Cushnie TTP, Lamb AJ (2005). Antimicrobial activity of flavonoids. Int J Antimicrob Agents.

[39] Cushnie TTP, Lamb AJ (2011). Recent advances in understanding the antibacterial properties of flavonoids. Int J Antimicrob Agents.

